# Airway stenosis secondary to mediastinal lymph node metastasis of lung adenocarcinoma treated with AERO stent and osimertinib: A case report

**DOI:** 10.1002/rcr2.1383

**Published:** 2024-05-14

**Authors:** Yuki Takigawa, Ken Sato, Tomoyoshi Inoue, Akiko Sato, Yui Furutaguchi, Mayu Goda, Keisuke Shiraha, Miho Fujiwara, Suzuka Matsuoka, Sho Mitsumune, Hiromi Watanabe, Kenichiro Kudo, Keiichi Fujiwara, Takuo Shibayama

**Affiliations:** ^1^ Department of Respiratory Medicine NHO Okayama Medical Center Okayama Japan

**Keywords:** AERO stent, airway stenosis, EGFR‐TKI, osimertinib

## Abstract

A woman in her mid‐50s was admitted to our hospital with airway stenosis secondary to mediastinal lymph node enlargement. An AERO stent was placed under rigid bronchoscopy. Immediately after stent placement, tissue sampling was performed on the lymph nodes. Metastatic lesions were found to have an *EGFR* mutation (exon 19 deletion). Consequently, osimertinib treatment was initiated 15 days after stent placement. The tumour partially responded to osimertinib, and the airway stenosis improved. The patient underwent stent removal 66 days after stent placement. Our findings indicate that temporary oncological emergencies due to airway stenosis may be bridged by airway stenting.

## Graphical abstract

A woman in her mid‐50s was admitted to our hospital with airway stenosis secondary to mediastinal lymph node enlargement and the AERO stent was placed under rigid bronchoscopy. Metastatic lesions were found to have an EGFR mutation. The tumour achieved a partial response to osimertinib, and the patient underwent stent removal.

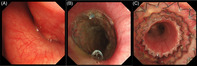

## INTRODUCTION

Airway stent placement is an established procedure for the treatment of airway disorders, such as stenosis and fistulas. The AERO stent is a fully covered hybrid self‐expandable metallic stent, which was approved in Japan in 2014. This stent can be safely removed from patients who require stent removal. AERO stent placement is both effective and safe for malignant airway disorders.^1^, ^2^ Because an AERO stent can be removed after stenosis improves, it can be used as a bridging therapy for anticancer treatment. The number of indications for molecular‐targeted agents in lung cancer treatment is increasing. Oncological emergencies due to airway disorders may be primarily overcome using AERO stents, allowing for prolonged survival using molecular‐targeted drugs.

## CASE REPORT

**FIGURE 1:**
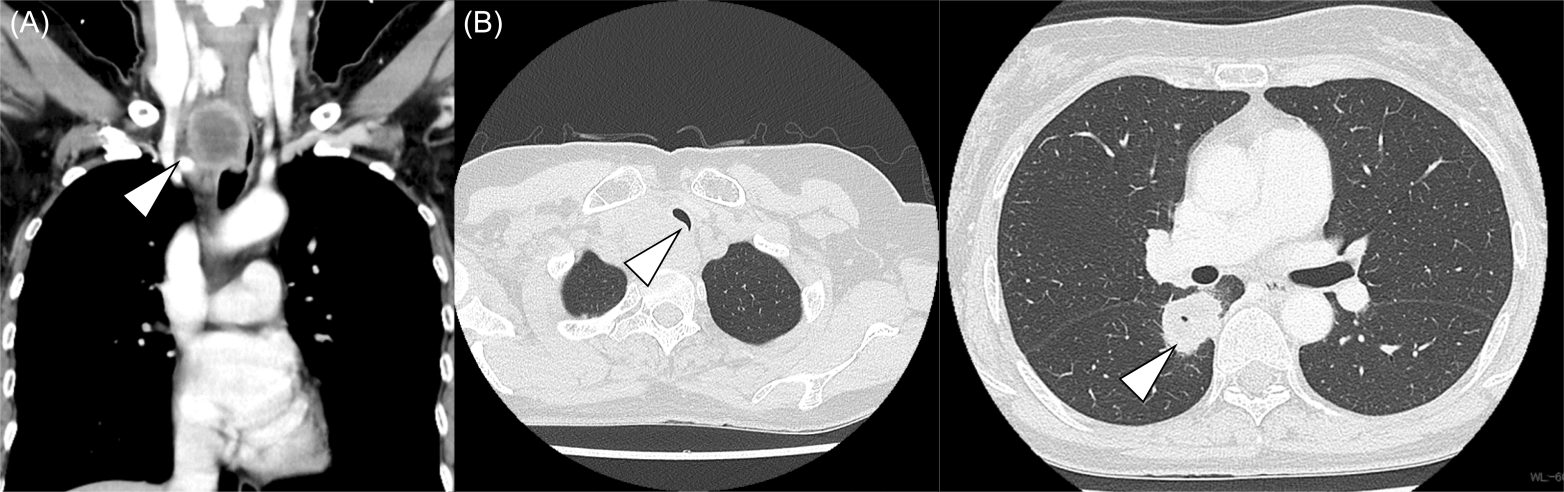
Contrast‐enhanced computed tomography (CE‐CT) on admission. (A) CE‐CT shows enlarged lymph nodes and stenosis of the upper‐to‐mid trachea (white arrow). (B) Chest CT shows a 3‐cm mass in the S6 segment of the right lower lobe.

A woman in her mid‐50s was referred to our hospital. She was a past smoker. Computed tomography (CT) showed enlargement of the right mediastinal lymph node (LN) #2R and a 3 cm‐sized mass in S^6^ of the right lower lobe (Figure 1). Hospitalization was scheduled for the following week. Four days later, she was admitted to the emergency department with strong dyspnea and difficulty to walk. Oxygen saturation was maintained in room air, but strong stridor was heard during auscultation. CT showed a risk of complete airway obstruction due to airway stenosis caused by the enlarged LN. The pulmonary intervention team discussed the present case and decided to perform emergency tracheal stent placement. Since the lesion was in the upper to mid trachea and no definitive diagnosis had been made, placement of an AERO stent (Merit Medical Systems, South Jordan, UT, USA) that could be removed after anticancer treatment was planned.

**FIGURE 2:**
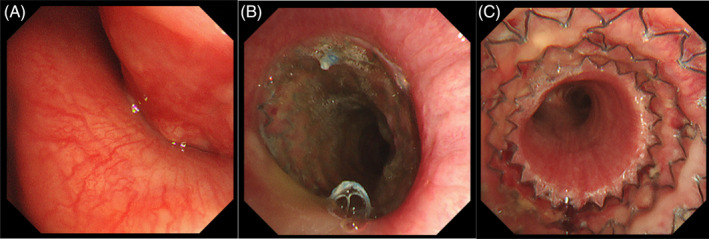
(A) Bronchoscopic image obtained before stenting in the patient. No airway invasion but stenosis due to extrinsic compression was observed. (B,C) Bronchoscopic image obtained after AERO stent placement.

Rigid bronchoscopy (EFER BRONCHOSCOPE; Harada Corporation, Osaka, Japan) was performed under general anaesthesia. A therapeutic flexible bronchoscope (BF‐1TQ290, 3.0‐mm working channel; Olympus Corporation) was then advanced into the upper trachea, which was almost completely obstructed from the right side of the tracheal wall, making peripheral observation with therapeutic bronchoscope beyond the stenosis impossible (Figure 2A). Following balloon dilation of trachea, an AERO stent (16 × 60 mm) was placed under the overwire system without any complications (Figure 2B,C). Tissue sampling of LN #2R via percutaneous ultrasound‐guided needle biopsy was performed three times using the same procedure. Two days later, a trans‐bronchial biopsy of the right lower lobe mass was performed with an ultrathin bronchoscope (BF‐MP290F, 1.7‐mm working channel; Olympus Corporation) using a radial EBUS probe. Pathological findings of the lung mass in the right lower lobe and LN revealed lung adenocarcinoma and LN metastasis, respectively.

**FIGURE 3:**
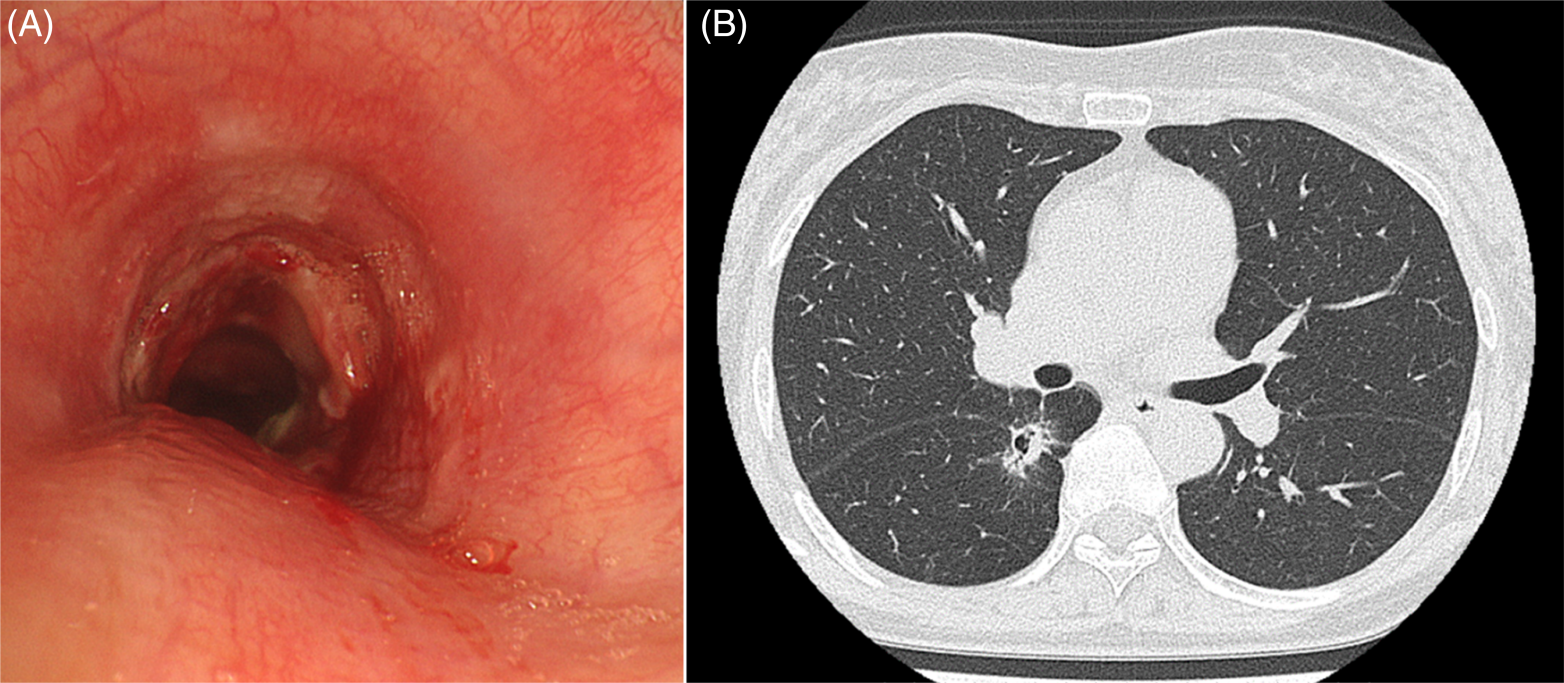
(A) Bronchoscopic image obtained after AERO stent removal. (B) Chest CT shows a mass in the S6 segment of the right lower lobe successfully treated with osimertinib.

Based on imaging findings, the patient was diagnosed with cT2aN2M1b stage IVB. The tumour harboured an *EGFR* exon 19 deletion mutation based on Amoy Dx® (Amoy Diagnostics, Xiamen, China), which was revealed 10 days after emergency department admission. Osimertinib treatment was initiated 15 days after stent placement. The tumour partially responded to osimertinib, and the airway stenosis improved. On day 66 after placement, the stent was removed without complications (Figure 3A,B).

## DISCUSSION

Osimertinib was approved as a first‐line treatment for *EGFR*‐mutation positive advanced non‐small cell lung cancer (NSCLC) in Japan in 2018. The median overall survival was 38.6 months in the osimertinib group.^3^ However, in advanced cancer, the development of airway stenosis or other oncological emergencies may result in death, and anticancer therapy may not be initiated in some cases. As in the present case, the diagnosis was not made prior to airway stenting; therefore, a specimen from the malignant tumour should be obtained, and multigene panel testing for cancer should be performed as soon as possible. In this case, stenting was performed on the day of admission, and we detected *EGFR* exon 19 deletion only 10 days after emergency admission.

The AERO stent is a fully covered hybrid self‐expandable metallic stent approved in Japan in 2014. These stents can also be safely removed from patients who require stent removal, similar to silicone stents.^1^, ^2^ Few similar reports exist on combination therapy with molecular‐targeted agents and an airway stents.^4^, ^5^, ^6^, ^7^ It has been reported that airway stenosis in an ALK‐positive lung cancer case with severe airway stenosis improved after ALK‐TKI treatment and temporary placement of a silicone Y‐stent, which was removed 5–6 months later.^5^, ^6^ Furthermore, there have been reports on AERO stent removal due to the effects of immune checkpoint inhibitors.^2^ Bridging therapy with airway stenting will become increasingly important as the indications and types of molecularly targeted drugs and immune checkpoint inhibitors increase. Silicone and AERO stents, which are removable, have good indications as anticancer agents with high response rates. If a metallic stent is placed in such cases, it may not be possible to remove it, even if postoperative anticancer therapy achieves a good response. Mechanical ventilation under intubation or extracorporeal membrane oxygenation is an alternative to airway stenting. If airway stenting is not possible, the patient should be carefully watched in the intensive care unit. Mechanical ventilation should be initiated when the risk of complete airway obstruction becomes higher. EGFR‐TKI has been reported to improve the survival of critical EGFR‐mutant lung cancer patients undergoing mechanical ventilation.^8^ Therefore, the present case could have been treated with EGFR‐TKI, even if she was on a mechanical ventilator without airway stenting. However, the EGFR mutation was not known at the time of admission, and ventilator placement for patients with advanced lung cancer should be carefully considered. In this case, stenting was the appropriate choice because the patient's performance status improved and was able to walk and eat the day after AERO stent placement.

Amoy Dx® was reported to have a shorter turnaround time (TAT), higher success rate, and detection rate than next‐generation sequencing (NGS) panels.^9^ While other NGS methods require more than 2 weeks to reveal results, the TAT of AMOY was only 5 days. In the present case, a histopathological diagnosis was made 4 days after AERO stenting, followed by AMOY examination the next day, and the TAT was 5 days. In cases of oncological emergencies, AMOY with a short TAT is recommended because of the need for early treatment.

Although overall survival after AERO stent placement was reported to be 2.7–3.3 months,^1^, ^2^ this patient had already survived more than 6 months at the time of the submission of this report, and her performance status was 0. Approximately 30% of lung adenocarcinomas in Japanese patients lack driver oncogene aberrations,^10^ and few cases can be treated with molecularly targeted drugs. Depending on the driver oncogene aberrations in each patient, airway disorders may be primarily overcome using airway stents, and survival may be prolonged using molecular‐targeted drugs.

In conclusion, temporary oncological emergencies due to airway stenosis may be bridged by airway stenting and additional treatments to improve patient prognosis.

## AUTHOR CONTRIBUTIONS

Yuki Takigawa and Ken Sato wrote the manuscript, which was reviewed by all co‐authors. All authors have approved the final version for submission.

## CONFLICT OF INTEREST STATEMENT

None declared.

## ETHICS STATEMENT

The authors declare that appropriate written informed consent was obtained for the publication of this manuscript and accompanying images.

## Data Availability

The data that support the findings of this study are available from the corresponding author upon reasonable request.
